# Reaching the Unreachable: Providing STI Control Services to Female Sex Workers via Mobile Team Outreach

**DOI:** 10.1371/journal.pone.0081041

**Published:** 2013-11-25

**Authors:** Pablo E. Campos, Anne L. Buffardi, César P. Cárcamo, Patricia J. García, Clara Buendia, Marina Chiappe, Geoff P. Garnett, Ana Maria Xet-Mull, King K. Holmes

**Affiliations:** 1 Epidemiology, STD/AIDS Unit, School of Public Health and Administration, Universidad Peruana Cayetano Heredia, Lima, Peru; 2 Center for AIDS & STD, University of Washington, Seattle, Washington, United States of America; 3 Department of Infectious Diseases Epidemiology, Imperial College, London, United Kingdom; 4 Department of Global Health, University of Washington, Seattle, Washington, United States of America; 5 Department of Medicine, University of Washington, Seattle, Washington, United States of America; Rollins School of Public Health, United States of America

## Abstract

**Background:**

As part of a community-randomized trial of a multicomponent intervention to prevent sexually transmitted infections, we created Mobile Teams (MTs) in ten intervention cities across Peru to improve outreach to female sex workers (FSW) for strengthened STI prevention services.

**Methods:**

Throughout 20 two-month cycles, MTs provided counseling; condoms; screening and specific treatment for Neisseria gonorrhoeae (NG), Chlamydia trachomatis (CT), and vaginal *Trichomonas vaginalis* (TV) infections; and periodic presumptive metronidazole treatment for vaginal infections.

**Results:**

MTs had 48,207 separate encounters with 24,814 FSW; numbers of sex work venues and of FSW reached increased steadily over several cycles. Approximately 50% of FSW reached per cycle were new. Reported condom use with last client increased from 73% to 93%. Presumptive metronidazole treatment was accepted 83% of times offered. Over 38 months, CT prevalence declined from 15·4% to 8·2%, and TV prevalence from 7·3% to 2·6%. Among participants in ≥9 cycles, CT prevalence decreased from 12·9% to 6·0% (p <0·001); TV from 4·6% to 1·5% (p <0·001); and NG from 0·8% to 0·4% (p =0·07).

**Conclusions:**

Mobile outreach to FSW reached many FSW not utilizing government clinics. Self-reported condom use substantially increased; CT and TV prevalences declined significantly. The community-randomized trial, reported separately, demonstrated significantly greater reductions in composite prevalence of CT, NG, TV, or high-titer syphilis serology in FSW in these ten intervention cities than in ten matched control cities.

## Introduction

Sex workers experience high risk for acquisition and transmission of sexually transmitted infections (STIs)[1]. Mathematical modeling suggests condom promotion and repeat STI screening and treatment of female sex workers (FSW) can reduce HIV incidence and prevalence among FSW[2] and lower population-level STI prevalences[3].

Interventions employing both strategies have achieved success[4] increasing FSW condom use across all studies[5-16] and decreasing STI in most studies, although the impact on specific STI has varied, particularly for Chlamydia trachomatis (CT) infection[5,6,8-12,14-17]. Most studies have focused on women attending clinics [5,8]; a few increased service outreach to improve coverage [6,7].

The Peruvian National STI/HIV Control Program offers periodic STI screening and treatment for FSW through government clinics throughout the country. A 1991-1992 evaluation found no significant differences in prevalences of Neisseria gonorrhoeae (NG), CT, or syphilis between FSW receiving or not receiving these services[7]. Services revamped in Lima to include improved screening, free treatment, counseling and free provision of condoms, led to significant decreases in prevalences of NG, CT, *Trichomonas vaginalis* (TV), and bacterial vaginosis, and significantly increased condom use[18].

We then undertook a national trial of STI prevention and control in Peru (the Peru PREVEN Study), randomizing 20 cities in pair-wise fashion with 10 receiving a multicomponent STI control intervention, and 10 receiving status quo services[19]. The objective of the trial was to reduce the composite prevalence of CT, NG, TV, or syphilis seropositivity among FSW, and among the general population. This paper aims to describe one of the intervention components of the Peru PREVEN Study, the Mobile Teams (MTs) to improve outreach, deliver STI-related services to FSW, especially to those not already accessing government clinics.

## Materials and Methods

Initial community mapping of sex work venues included brothels, bars, nightclubs, street-based venues and truck stops. From November 2002 to April 2003 we conducted baseline surveys of STI prevalences, demographics, and behaviors of FSW in 24 cities[20], including the 20 subsequently chosen for the randomized intervention trial. The MT intervention for FSW then took place in the 10 intervention cities from July 2003, through 20 outreach cycles, each lasting eight weeks, with interventions and final outcome prevalence surveys completed in December 2006. Sex work venue mapping continued throughout the intervention, as venues closed, relocated, or newly emerged.

### PREVEN MT Structure and Activities

Each MT included an FSW peer educator and a nurse or midwife with experience providing clinical services to FSW. All MT members attended an initial three-day group training in Lima, with subsequent group training annually. Supervisors visited each MT at least every six months. Each MT maintained email and phone contact with national and regional PREVEN coordinators.

During every cycle, MTs traveled to all identified sex work venues on Thursday, Friday, and Saturday evenings, when venues were open with maximum numbers of FSW working. Identifiable by blue PREVEN smocks, MT members carried supplies, collecting and placing specimens into portable ice-gel coolers. Interaction spaces varied, from private bedrooms in brothels to small side rooms at bars.

At MT encounters, FSW provided verbal consent and self-obtained vaginal swab (SOVS) specimens including one polyester swab for polymerase chain reaction (PCR) testing, and one cotton swab for TV culture. Health workers then conducted educational counseling and motivational interviewing, promoting consistent, correct condom use with commercial and non-commercial partners, and use of local government STI clinic services. Women initially received 10 condoms per cycle, increasing to 50 per cycle in July 2005, with increased government condom supply. Non-pregnant women not breast-feeding and able to avoid alcohol for 72 hours were offered periodic presumptive treatment (PPT) with 2 g oral metronidazole at each cycle. From February 2005 through December 2006, a laboratory technician joined each MT, and every 16 weeks offered onsite testing of fingerstick whole blood using the Abbott Determine Syphilis TP^TM^ test, providing results within 15 minutes, and referring seropositive FSW to local government clinics for rapid plasma reagin (RPR) testing and free treatment per national guidelines[21].

Each encounter with FSW at the worksite lasted approximately 15-20 minutes. In attempting to distinguish new from continuing participants, each participant was given a unique study code (also linked to test specimens) at each visit, and was asked if she had previously participated and had a previous visit code. 

MT returned the following week to deliver test results; and provide free treatment with ciprofloxacin 500 mg orally for a positive NG test, azithromycin 1 gm orally for a positive CT test, and metronidazole 2gm orally for a positive TV culture for those not already presumptively treated with metronidazole. Nine of ten cities consistently recorded treatment coverage for positive tests. For cycles 2 to 20, coverage was 62% for NG, 67% for CT, and 86% for TV. Interviews about adverse reactions to metronidazole began with an open-ended question, then probed for specific symptoms. Visiting on average three to five venues each night, MTs repeated this two-visit process until all venues had received initial and follow-up visits throughout each implementation cycle.

### Laboratory Testing

At sex work venues, the cotton SOVS from each FSW was inoculated into BioMed InPouch™ TV for culture, and the polyester one into a cryovial for centralized PCR testing in Lima for NG and CT using Roche COBAS® AMPLICOR CT/NG. Each evening, specimens were taken to local government laboratories for TV culture incubation and temporary storage of cryovials at −20°. TV cultures were examined locally daily for five days for motile trichomonads. Cryovials were shipped weekly on ice to the central laboratory in Lima where 2-sucrose phosphate medium was added and PCR testing performed. Results emailed weekly from central and local labs to the local MT, allowed specific treatment within one week of specimen collection. To assess trends in prevalences of NG, among women with ≥9 MT encounters, the GenProbe Aptima Combo 2 Assay was used for NG confirmation of specimens with an AMPLICOR NG OD ≥3·5, because of reported low-specificity of the AMPLICOR assay for NG[22,23].

The laboratory manager from Lima visited each local lab every six months. She identified three cities where local TV culture results were not correlated with centralized quality control by GenProbe Aptima (TV) Assay, whereas nearly 100% of positive cultures for the remaining seven laboratories were confirmed by Aptima TV Assay. Laboratory technicians from the three non-conforming laboratories were retrained in July-August 2005 and closely monitored thereafter. Since TV test results from these three cities were considered invalid for the initial phase of the intervention, we present TV results for the remaining seven cities only.

### Statistical Methods

We used SPSS 12·0·1 (Chicago, IL), SAS 9·2 (Cary, NC), and Stata 8·2 (College Station, TX) for statistical analyses, calculated descriptive and chi-square statistics, and performed linear and logistic regression analyses. Multivariate analyses used backwards elimination, beginning with all variables significant in univariate analyses at p <0·10. Using random effect models, we evaluated changes in prevalence across time, accounting for repeated measures. We present results grouped for: 1) all MT encounters (n=48,245) and 2) individual FSW, regardless of the number of encounters (total number of FSW=24,814).

### Ethics Statement

Institutional review boards from the University of Washington Human Subjects Division, from the Universidad Peruana Cayetano Heredia, and from the US Naval Medical Research Unit – 6 in Lima Peru, approved protocols, consent forms and procedures, and instruments. The IRBs waived the need for written informed consent from FSW to protect anonymity (Code of Federal Regulations, Title 45, 46.116 c). We obtained a waiver of parental or guardian permission for minor FSWs (between 16 and less than 18 years old), due to the special circumstances in which parents were unavailable and the participation offered benefits (health services and prevention) at minimum risk. Women were approached at FSW venues and were invited to participate. All FSW participating provided verbal informed consent and received a copy the information statement. FSW received free condoms, HIV and STI tests, and treatment for STI.

## Results

### Acceptability & Participation

**Figure 1:**
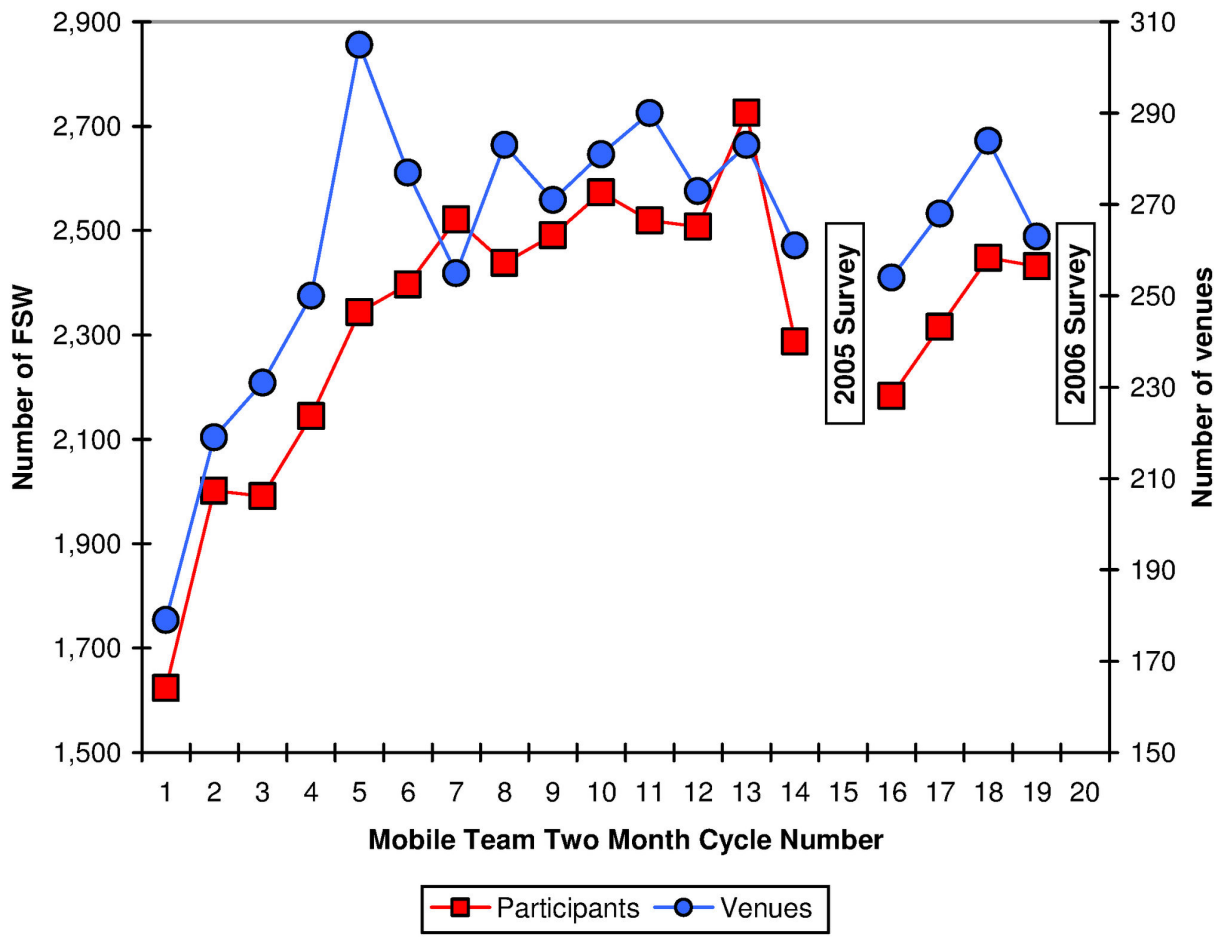
Numbers of female sex workers and commercial sex venues reached by Mobile Teams. The numbers of female sex workers and the numbers of commercial sex venues reached by mobile teams during the 20 eight-week intervention cycles is represented here. The numbers of sex work venues reached per cycle increased steadily from the first intervention cycle to the fifth cycle, then leveled off. Data from cycles 15 and 20 can’t be disaggregated from data for the 2005 and 2006 surveys of random samples of FSW in the ten cities, and therefore are not presented here.

Venue administrators accepted PREVEN MT activities; 99% of FSW approached agreed to participate. Numbers of sex work venues reached per cycle increased steadily from 171 in the first intervention cycle to 281 in the fifth cycle, then leveled off. FSW participation increased from 1,624 women in cycle 1, leveling off after cycle 7, with 2,432 women reached in cycle 19 (Figure 1), indicating increased numbers of FSW receiving services, and numbers of sex venues reached over time.

**Figure 2:**
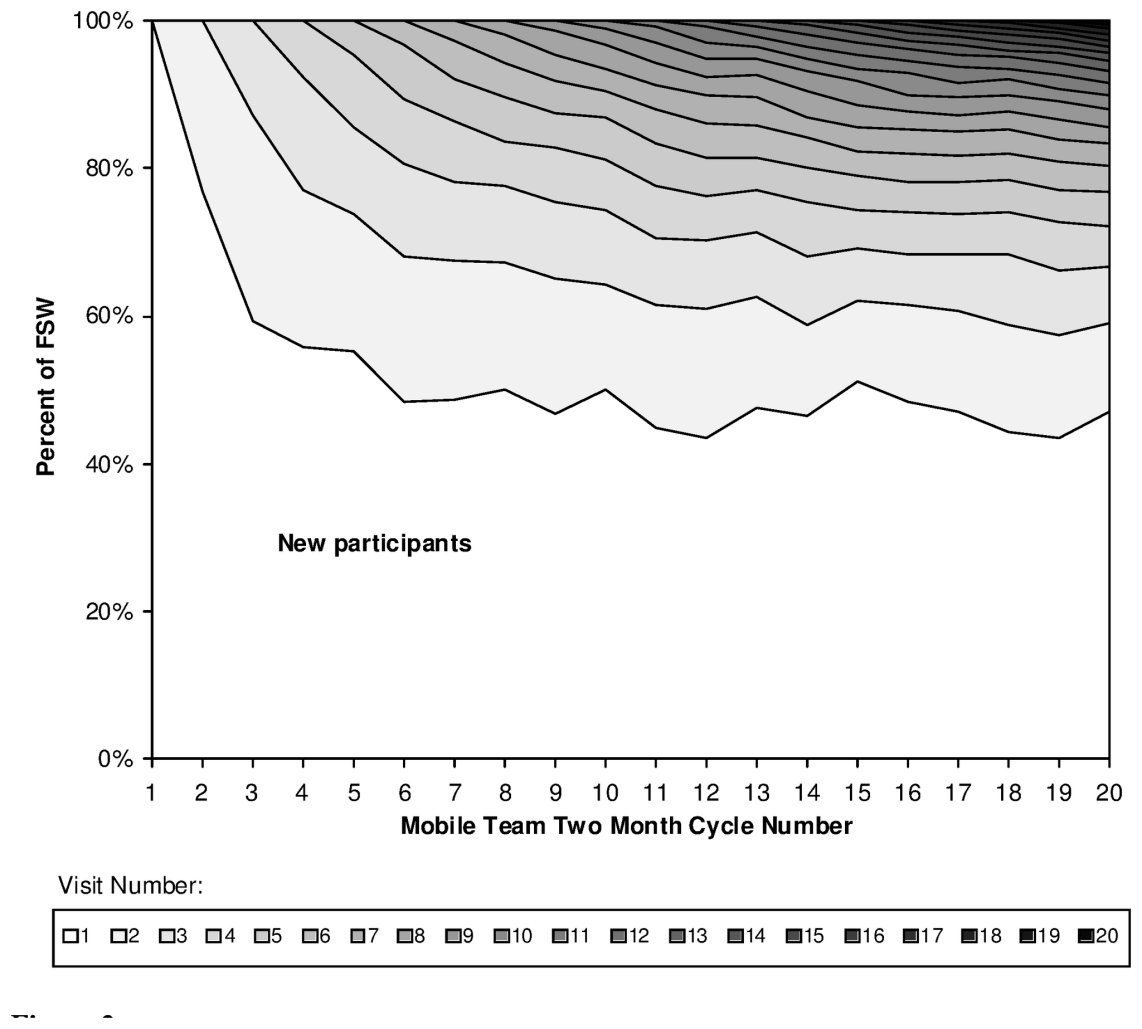
Proportion of female sex workers new to Mobile Teams at each intervention cycle. For each cycle, the number of encounters decreases from top to bottom of the figure; participants with greatest number of Mobile Team encounters are depicted at the top, those with lowest (i.e., new participants) at the bottom.

Approximately half of FSW for each cycle after cycle 5 were identified as first time participants (Figure 2), potentially attributable to mobility, short duration of sex work, or, intermittent sex work (e.g., when not menstruating or when income was needed). Also, because names were not collected, it was difficult to identify some returning FSW as previous participants. Of 29,835 encounters with FSW reporting having being approached in previous cycles, in 49.7% it was possible to determine the code assigned to the previous encounter. 

The median (mean) duration of sex work reported by 4,100 FSW in the baseline survey was 24 (43·4) months (SD 56·74 months, range 0–600 months); 43% had worked for ≤12 months; 40% reported working in at least one other city in the past year. Although 17,166 FSW had only one encounter with the MT, the remaining 7,648 reported a mean of 4·1 encounters (median 3, range 2-20). Numbers of MT encounters were positively associated with older age, previous use of government clinics, and nightclub or brothel work, and negatively associated with working in a bar or street-based sex work (p <0·001).

Table 1 presents demographic and behavioral characteristics and STI prevalences among FSW at first MT encounter. Ages ranged from 14-72 years; 44% worked in Andean cities, 17% in the Amazonian jungle, and 39% in coastal cities, reflecting numbers of cities from each location selected for the intervention.

**Table 1:** Demographic & behavioral characteristics and STI prevalences among 24,814 Peruvian FSW receiving PREVEN Mobile Team services for the first time.

	**n**	**%**
Age (n=24,742)		
<18	1,454	5·9%
18-20	6,080	24·6%
21-24	7,022	28·4%
25-29	5,017	20·3%
>29	5,169	20.9%
Region (n=24,814)		
Coast	9,776	39·4%
Andes	10,953	44·1%
Jungle	4,085	16·5%
Sex work venue (n=23,564)		
Brothel	4,233	18·0%
Bar	6,640	28·2%
Night club	9,449	40·1%
Street	2,716	11·5%
Other	531	2·3%
Previous government STI clinic-based medical exam ever (n=23,990)	9,358	39·0%
Reported using condom with last client (n=24,591)	19,325	78·6%
Accepted presumptive metronidazole treatment (n=24,802)	19,240	77·6%
STI prevalences		
Chlamydial infection* (n=23,065)	3,114	13·5%
Trichomoniasis** (n=16,424)	704	4·3%
Gonorrhea* (n=21,224)	794	3·7%

* Positive by Roche AMPLICOR PCR

** Positive by InPouch TV culture (data limited to 7 cities with satisfactory *T. vaginalis* culture quality assessment throughout the study).

### Metronidazole Treatment

Presumptive metronidazole treatment was ever accepted by 20,209 (81%) of 24,814 women, at 81% of 48,245 times offered. Acceptance ranged from 65% of Andean women to 88% in the jungle, to 95% on the coast (p <0·001) and increased from 77% in the first encounter to over 90% after the 8^th^ cycle. Among those declining, 80% couldn’t avoid consuming alcohol for 72 hours (alcohol consumption with customers in bars or clubs was expected), 8% were pregnant, 3% breastfeeding, 3% cited allergies, 2% experiencing delayed menstruation, and 5% offered no reasons. Among 20,209 women ever accepting metronidazole, 6,926 (34%) spontaneously reported at least one adverse reaction, including nausea (34%), headache (23%), vomiting (11%), abdominal pain (11%), or metallic taste (11%); only two reported tingling or numbness.

### Trends in STI Prevalences

**Figure 3:**
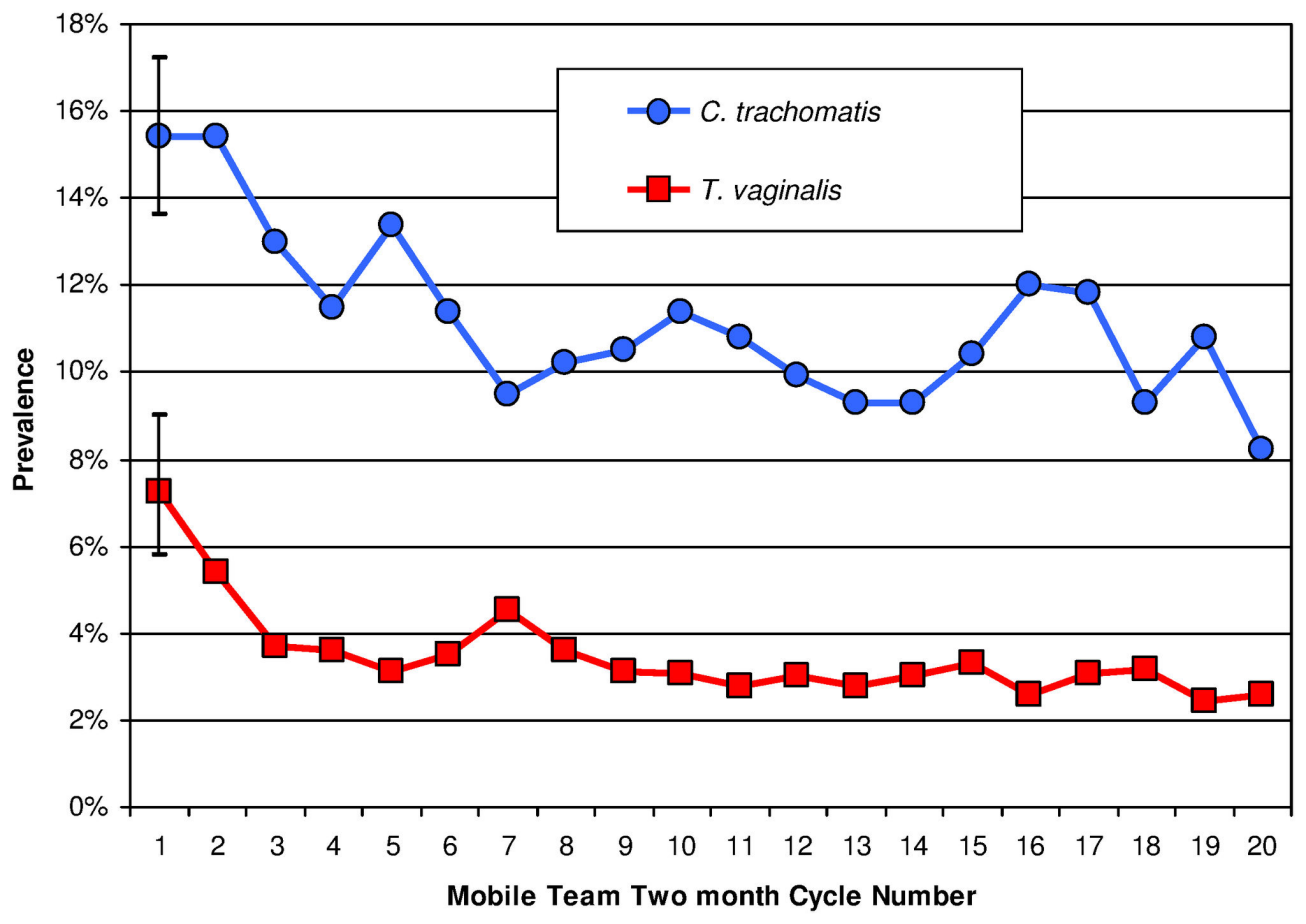
Prevalences of *C. trachomatis* and *T. vaginalis* infections among female sex workers. The 95% confidence intervals are shown for Cycle 1 (when the number of encounters was smallest). Reductions in prevalences are significant for *C. trachomatis* (p <0·001) and for *T. vaginalis* (p <0·001).

During the first cycle, 15·4% of FSW tested PCR-positive for CT, declining to 8·2% by the final cycle (p <0·001); 7·3% tested culture-positive for TV, declining to 2·6% (p <0·001, Figure 3); 4·0% tested AMPLICOR PCR positive for NG, varying from 2·6% to 5·1% thereafter. First cycle prevalences across cities ranged widely from 2·5% to 27·5% for CT, from 0·9% to 7·4% for NG, and, for the seven cities included in TV analyses, from 1·5% to 11·7% for TV. Over the three-year intervention, CT and TV prevalences decreased steadily, but NG prevalence in SOVS by PCR assay did not.

**Figure 4:**
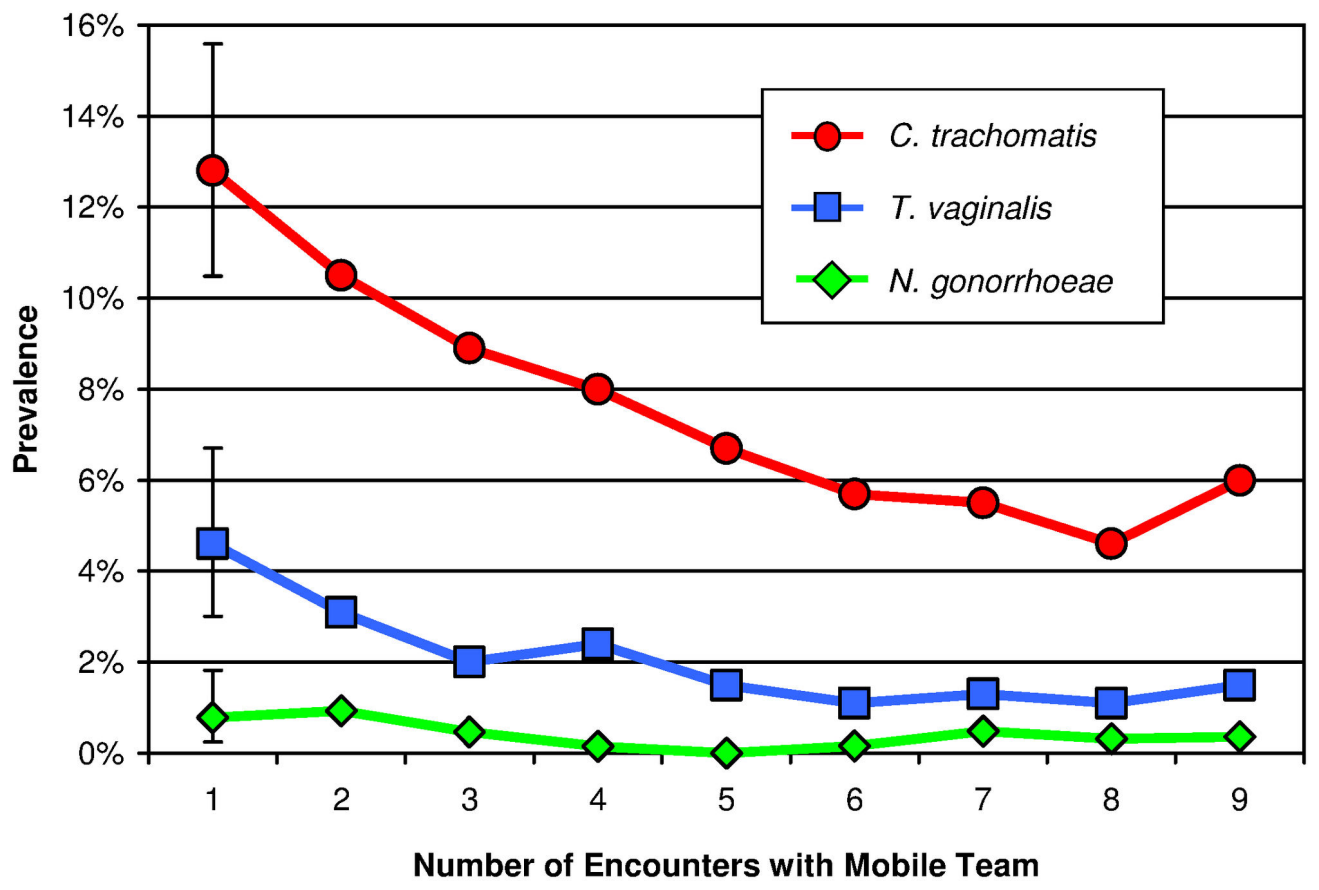
Prevalences of *C. trachomatis*, *N. gonorrhoeae*, and *T. vaginalis* among sex workers. The prevalences are presented in relation to the number of encounters with the Mobile Team. Analysis is restricted to 714 sex workers with at least nine encounters with the Mobile Team. 95% confidence intervals are shown for the first encounter. Reductions in prevalences are significant for *C. trachomatis* (p <0·001) and *T. vaginalis* (p <0·001), but not for *N. gonorrhoeae* (p =0·07). *N. gonorrhoeae* infection was defined by AMPLICOR NG OD ≥3·5, confirmed by positive Aptima Combo2 assay.

To control for participation bias, we examined CT, TV, and NG prevalence trends among FSW with nine or more MT encounters. Over nine encounters, CT prevalences declined from 12·9% to 6·0% (p <0·001); NG (AMPLICOR OD ≥3·5, confirmed by Aptima assay) from 0·8% to 0·4% (p=0·07); and TV from 4·6% to 1·5% (p <0·001, Figure 4). We couldn’t monitor trends in RPR positive, TP•PA confirmed serologies which were performed at separate government clinics.

### Association of STI Prevalences with Demographic and Behavioral Characteristics and Prior Testing

At first MT encounters, Andean or jungle residence (compared to coastal residence), and bar or night club work (compared to brothel work) were significantly associated with CT and with TV; working in the streets was associated with TV (Table 2); self-reported condom use with last client, and having undergone a previous government clinic STI exam were negatively associated with these two infections (p <0·001 for each comparison). Previous STI clinic exam and condom use with last client did not seem to protect against gonorrhea in this study. For all encounters, having received presumptive metronidazole treatment was negatively associated with TV infection (adjusted OR 0·79, 95% CI 0·66 - 0·96, p <0·001); and number of previous MT encounters was negatively associated with CT (adjusted OR 0·91, 95% CI 0·89 - 0·92, p <0·001) and TV (OR 0·91, 95% CI 0·88 - 0·94, p <0·001).

**Table 2:** Multivariate logistic regression analyses of factors associated with detection of chlamydial infection and trichomoniasis by PCR testing at the First Mobile Team encounter with Peruvian female sex workers.

	Chlamydial infection (n=40,926) *				Trichomoniasis (n=30,733) *		
	AOR	95% CI			AOR	95% CI	
Age							
14-17	Ref				Ref		
18-20	0·90	0·79	1·03		0·70	0·55	0·90
21-24	0·68	0·60	0·78		0·51	0·40	0·67
25-29	0·52	0·45	0·60		0·47	0·36	0·62
>29	0·38	0·32	0·44		0·64	0·49	0·83
Region							
Coast	Ref				Ref		
Andes	1·58	1·44	1·74		2·33	1·89	2·87
Jungle	2·38	2·15	2·63		3·50	2·90	4·24
Sex work venue							
Brothel	Ref				Ref		
Bar	1·56	1·40	1·75		2·31	1·78	2·98
Night club	1·33	1·19	1·49		1·49	1·13	1·96
Street	1·02	0·87	1·20		2·11	1·55	2·86
Other	1·05	0·80	1·39		1·64	0·96	2·79
Previous STI clinic exam ever	0·89	0·83	0·96		0·56	0·49	0·65
Condom use with last client	0·77	0·71	0·84		0·60	0·50	0·71

* Analysis limited to those for whom *C. trachomatis* PCR results are available; and *T. vaginalis* culture results are limited to the 7 cities with satisfactory quality assurance on *T. vaginalis* cultures throughout the entire study.

AOR: Odds ratios adjusted for the other variables in the multivariate model presented.

### Sexual Risk Behaviors

In the first cycle, 73% reported condom use with last client, increasing to 93% by the final cycle; and across all cycles, was initially reported by 79% of all first time participants. For FSW having their first MT encounter, self-reported condom use with last client ranged from 66% of women in the Andes to 76% in the jungle to 94% on the coast (p <0·001). Self-reported condom use with last client was associated (p <0·001) with older age, previous clinic-based medical exam, later intervention cycle and number of MT encounters. For FSW with nine or more MT encounters, reported condom use with last client increased from 86% at the first encounter to 96% at ninth encounter.

### Government STI Clinic Utilization

In the first intervention cycle, 65% reported ever receiving government STI clinic services. This proportion rapidly declined over subsequent cycles, stabilizing around 35% after the fourth cycle. Over the entire intervention period, only 39% of women at their first encounter reported a previous government STI clinic exam. Prior government clinic use varied by work venue and by region, from 68% of women working in brothels to only 33-34% of women working in night clubs, bars, or on the street (p <0·001); and from 51% in jungle cities to 40% on the coast to 33% in the Andes. Older age also predicted previous use of government clinic services.

## Discussion

We have shown that nationally coordinated, locally implemented MT outreach for STI screening and specific treatment services, metronidazole PPT, and risk reduction counseling with condom promotion and provision, provided an effective, feasible approach toward achieving STI control, complementing existing clinic-based services for a high risk, mobile populations. We observed significant declines in CT and TV prevalences; and among participants in ≥9 cycles, non-significant declines from initially low prevalences of NG as well.

The PREVEN intervention emphasized screening and specific treatment rather than PPT for NG and CT, because of low NG prevalences. We centralized nucleic acid amplification testing (NAAT) for NG and CT screening in this trial when preliminary decentralized antigen testing for CT and culture for NG proved difficult to implement in the ten local laboratories. Centralized NAAT was performed on nearly 50,000 specimens collected by MTs, and on comparable numbers of specimens from the 2002, 2005, and 2006 surveys, demonstrating the potential for centralized molecular testing for STIs (and presumably, for selected other conditions, such as tuberculosis).. We promoted syphilis screening at existing government clinics until point of care syphilis tests could be implemented in 2005; and did not employ syndromic management for genital ulcers because chancroid ulcers had become rare in Peru. MT-FSW encounters occurred at sex venues (where clinical exams were not feasible), so metronidazole PPT rather than syndromic management was offered for vaginal infections. Complementing screening and specific treatment for CT, NG, and TV, the PPT with metronidazole was inexpensive ($0.14/treatment), causing no recognized serious adverse effects. PPT usage for longer periods would require continued attention to potential neurotoxicity, and optimizing frequency of PPT.

FSW mobility and intermittent sex work probably limited our ability to get treatment to all women found infected, and together with infrequent condom use with non-paying partners help explain why even larger declines in TV and CT prevalences weren’t seen.

A systematic review through mid-2006 identified 28 interventions to prevent HIV/STI among FSW in resource-poor settings [4]. Several evaluated behavioral interventions combined with STI treatment [5,9,10,12-15,17]. Reviewers found no community-randomized trials, but noted evidence that “innovative clinical services may increase the coverage of dispersed and clandestine SWs;” they concluded risk-reduction counseling plus condom promotion were consistently associated with reduced HIV and/or other STI risk, and that failure of two RCTs to show an effect of PPT or regular screening on STI rates might be explained by type 2 error. The review suggested effects of PPT on STI rates are short-lived, and longer intervals between PPT may lessen effect on STI prevalence. An adequately powered RCT[17]. of azithromycin PPT in Kenya did lower rates of CT, NG, and (surprisingly) TV. A recent systematic review of HIV/STI prevention for FSW in China[24] found mixed impact of prevention on STIs in nine studies, and called for more rigorous study design.

A review[25] of six recent observational studies of PPT for FSW, most employing azithromycin combined with cefixime, found substantial declines in prevalence of CT and NG. Another study[26] of Kenyan FSW randomized to monthly PPT with oral metronidazole 2 g plus fluconazole 150 mg, versus placebos, achieved significantly fewer episodes of bacterial vaginosis and non-significant reductions of incident trichomoniasis and vaginal candidiasis in the treatment arm. The multicomponent package of STI services delivered to FSW in India’s large scale non-randomized Avahan intervention[27] produced evidence for impact on HIV, high titer syphilis, and CT or NG in Karnataka state[28]. So, rigorously conducted and adequately powered individual-randomized trials of STI control in FSW have demonstrated significant impact on CT, NG, and TV prevalence[17] and on bacterial vaginosis[26]. But we identified no community-randomized trials that examined the impact of comprehensive prevention interventions on STI prevalences in FSW.

In conclusion, time-series analyses presented here found that less than 40% of all FSW initially reached by the MT reported previous government STI clinic exams, and that counseling plus condom promotion, screening and treatment for CT, NG, and TV, plus metronidazole PPT significantly lowered prevalences of CT and TV in FSW. This underpins results of our 20-city randomized PREVEN trial, which showed a 33% reduction in composite relative risk of curable STIs (CT, NG, TV, or syphilis) in FSW in the ten intervention cities, compared with ten paired control cities[19]. Expanding health services coverage for FSW is a public health priority to reduce STI among FSW. In Peru, FSW in the jungle had highest rates of CT and TV infections, and of syphilis seroreactivity, and clearly warrant special attention.

The Pan American Health Organization Regional HIV/STI Health Sector Plan recommends enhanced access to prevention and care, and condom promotion and behavior-change communication for vulnerable groups including FSW. The plan advocates STI control as a potential method for preventing HIV transmission [29]. WHO recommendations also exist for research on PPT for STI in high-risk populations [30]; however, emerging antimicrobial resistance of *N. gonorrheae* to fluoroquinolones and recently to azithromycin, and the lessened NG susceptibility to third generation cephalosporins, should trigger an alarm about continued use of the PPT approach for gonorrhea. 

## Supporting Information

File S1 **Recruitment Script.** Document used by the mobile team members to inform FSW present in the work site about the study and invite them to participate.
(DOC)

File S2 **Consent Process Guide.** Document used by the mobile team members informed women about the purpose, benefits, procedures, and risk and discomfort that could occur as a consequence of their participation in the study.
(DOC)
