# Supplementary material for: Reaching the Unreachable: Providing STI Control Services to Female Sex Workers via Mobile Team Outreach
Source: PLoS One. 2013 Nov 25;8(11):e81041. doi: 10.1371/journal.pone.0081041 (PMC3839873; doi:10.1371/journal.pone.0081041)
Supplement: File S1 — Recruitment Script. Document used by the mobile team members to inform FSW present in the work site about the study and invite them to participate. (DOC) [file pone.0081041.s001.doc]

**Cayetano Heredia University – University of Washington - Imperial College**

**Urban Community randomization Trial of Sexually Transmitted Disease Prevention**

**FSW INTERVENTION- MOBILE TEAM**

**Recruitment Script**

Each day, the Mobile Team will visit commercial sex venues determined ahead of time. Each FSW present in the work site will be informed and invited to participate in the survey. One of the members of the mobile team will approach the FSW and introduce herself.

“Hello, I am ___________________________ from Cayetano Heredia University. We, in close coordination with the Ministry of Health, are conducting a study to control STD. Would you like to hear more about this activity?”

If NO,

“Thank you very much”

If YES,

### “We are asking for your participation in this study as a volunteer. This study will evaluate if screening and treating STD by a mobile team is a feasible and efficient method for STD control among female sex workers in 10 Peruvian cities. The benefit to you is that you will receive screening and treatment, if necessary, for gonorrhea and chlamydia, and presumptive treatment for bacterial vaginosis and trichomoniasis at no cost. The benefit to society is that this information will help us test new approaches to control STD.

If you agree to participate in this study, we will provide STD counseling and explain how to take a vaginal sample using a cotton swab. We will also offer you treatment in case you have bacterial vaginosis and trichomoniasis. The swab will be shipped to Lima to test for gonorrhea and chlamydia at a central laboratory. Next week, the mobile team will visit you again at your work site to give you your test results, provide new treatment, and additional STD counseling. The Mobile Team will encourage you to make an appointment at the STD clinic next month, and will visit your work site every two months. May we continue to provide you with information about this study?”

If NO,

“Thank you very much”

If YES,

**Provide detailed information about the study and obtain informed consent for enrollment.**
