# Supplementary material for: Reaching the Unreachable: Providing STI Control Services to Female Sex Workers via Mobile Team Outreach
Source: PLoS One. 2013 Nov 25;8(11):e81041. doi: 10.1371/journal.pone.0081041 (PMC3839873; doi:10.1371/journal.pone.0081041)
Supplement: File S2 — Consent Process Guide. Document used by the mobile team members informed women about the purpose, benefits, procedures, and risk and discomfort that could occur as a consequence of their participation in the study. (DOC) [file pone.0081041.s002.doc]

**Cayetano Heredia University – University of Washington - Imperial College**

**Urban Community randomization Trial of Sexually Transmitted Disease Prevention**

# FSW INTERVENTION-MOBILE TEAM

**Consent Process Guide**

**[to be read by interviewer]**

I am going to read this declaration of information, and if you want, here is a copy for you to read silently. You can keep this copy. This will let you know what will happen if you participate in this study. We will review information that will be useful as a participant in the study, including the purpose, benefits, procedures, and risk and discomfort that could occur as a consequence of your participation in this study. If you have any question as we review this document, please interrupt me. Once we finish reviewing this document and answering your questions, I will ask you if you are interested in participating in this study. All right, then let’s begin.

Information Statement

**Investigators:**

Pablo E. Campos MD, MPH. STD Unit, Cayetano Heredia University, Lima. Phone: 319-0028 (Lima).

Patricia M. Segura RN. STD Unit, Cayetano Heredia University, Lima. Phone: 319-0028 (Lima).

Marina A. Chiappe. STD Unit, Cayetano Heredia University, Lima. Phone: 319-0028 (Lima).

King K. Holmes MD, PhD. CFAR, University of Washington, USA. Phone: 001-206-731-3620 (USA).

For questions about the study, call Dr. Pablo Campos at 996-5806 (Lima).

## INVESTIGATORS STATEMENT

We are asking for your participation as a volunteer in a research study. Please read this form carefully. Feel free to ask questions about possible risks and benefits, your rights as a volunteer, and anything else about the research that is not clear. When all questions have been answered, you can decide if you want to be in the study or not. This process is called "informed consent."

### PURPOSE AND BENEFITS

This study is being done to determine if mobile teams that screen and treat sexually transmitted diseases (STD) among female sex workers in 10 Peruvian cities can help keep STD from spreading. The benefit to society is that this information will help us test new ways to keep people from getting STD. The benefits to you could include access to STD testing and medicine if necessary, for gonorrhea and chlamydia; and medicine that treats bacterial vaginosis and trichomoniasis at no cost.

#### PROCEDURES

If you agree to participate in this study, the mobile team -which includes a nurse midwife and a peer educator- will provide STD counseling and explain how to obtain a vaginal sample using a cotton swab. The team will also offer you medicine which treats bacterial vaginosis and trichomoniasis. The swab will be shipped to Lima to perform tests for gonorrhea and chlamydia at a central lab. During the next two weeks, the mobile team will visit you again at your work site to give you your test results, provide more medicine, if necessary, and additional STD counseling. The mobile team will encourage you to make an appointment at the STD clinic, and will visit your work site every two months.

We will also ask for your permission to store a small amount of your vaginal sample for future research. These might include research on herpes, *Mycoplasma genitalium*, chlamydia, gonorrhea and other germs that we learn about in the future.

#### RISKS, STRESS OR DISCOMFORT

Obtaining vaginal samples by yourself could produce a slight discomfort, but it does not have risk to your health. Drinking any amount of alcohol after the use of the medicine you receive (metronidazole) may cause you to feel tired, nauseous or vomit, and these could be severe. Alcohol should not be consumed for at least 72 hours after taking this medicine (metronidazole).

#### OTHER INFORMATION

The information we obtain will be kept confidential. Your name will not be written on any forms or sample labels. We will give you a card with your study code. You will need to keep this code to receive your test results during our next visit. We will greatly appreciate your help in participating in this study.

Do you have any question about the study, or would you like to discuss any part of it before we continue?

*[Pause for a few seconds to allow the participant think about a question]*

I know we reviewed a lot of information but I would like to make sure that you understand. Can you describe some of the risks of participating in this study? Can you tell me what you will have to do if you decide to participate in the study?

[*If the participant can not explain the risks or procedures of the study, you have to clarify the information about the risks and procedures of the study*.]

Now that you understand what your participation means in this study, do you want to participate?

- *No, I don’t want to participate in this study.*

We respect your decision not to participate. Thanks for your time.

- *Yes, I want to participate in this study.*

*[Continue]*

## Declaration of the participant

Since you agree, please repeat after me:

*The study described has been explained to me, and I volunteer to take part in this research. I had the opportunity to ask questions. If in the future I have questions about the study, these will be answered by one of the investigators listed above.* If I have questions about my rights as a participant in a research study, you can contact the Ethics Committee at Cayetano Heredia University (phone 01-3190005 ext. 2271) or you can contact by mail the University of Washington IRB, at Human Subjects Division Box 351412, Seattle WA 989195 USA.

I will get a copy of this consent form.

- Yes, I give permission for my vaginal sample to be stored for future research studies.
- Please throw out my vaginal sample after this study and do not use it in future research.

Signature of interviewer certifying that informed consent has been given verbally by respondent.

_______________________________ _______________________________ Date: ___ /___ /____

Interviewer’s signature Interviewer’s full name (day/month/year)

1. Participant

Investigator’s file
